# Supplementary figures and images for: Transovarial transmission of a core virome in the Chagas disease vector Rhodnius prolixus
Source: PLoS Pathog. 2021 Aug 18;17(8):e1009780. doi: 10.1371/journal.ppat.1009780 (PMC8372912; doi:10.1371/journal.ppat.1009780)

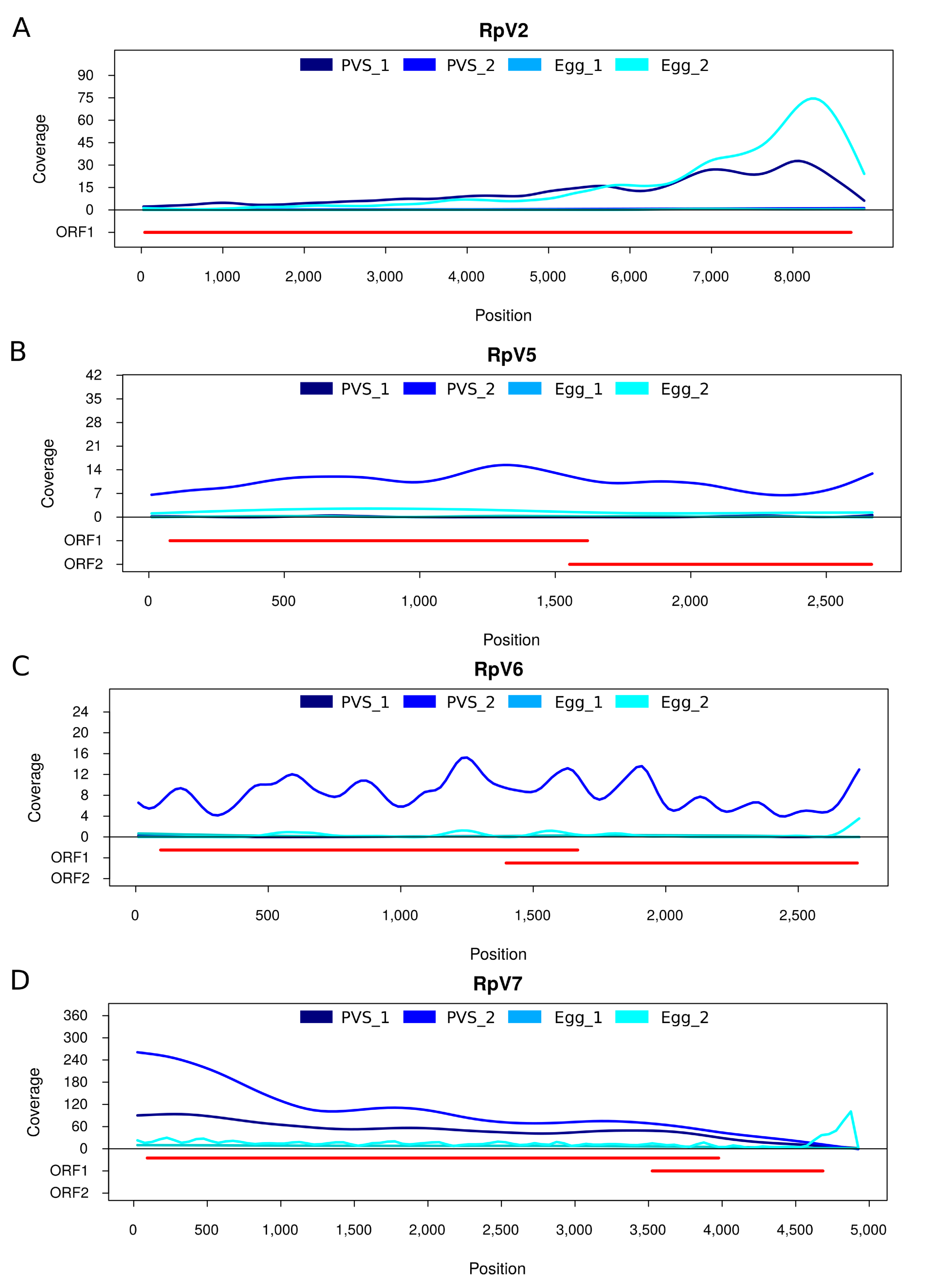

Supplement: S1 Fig — (A) RpV2, (B) RpV5, (C) RpV6 and (D) RpV7. (TIFF) [file ppat.1009780.s001.tiff]

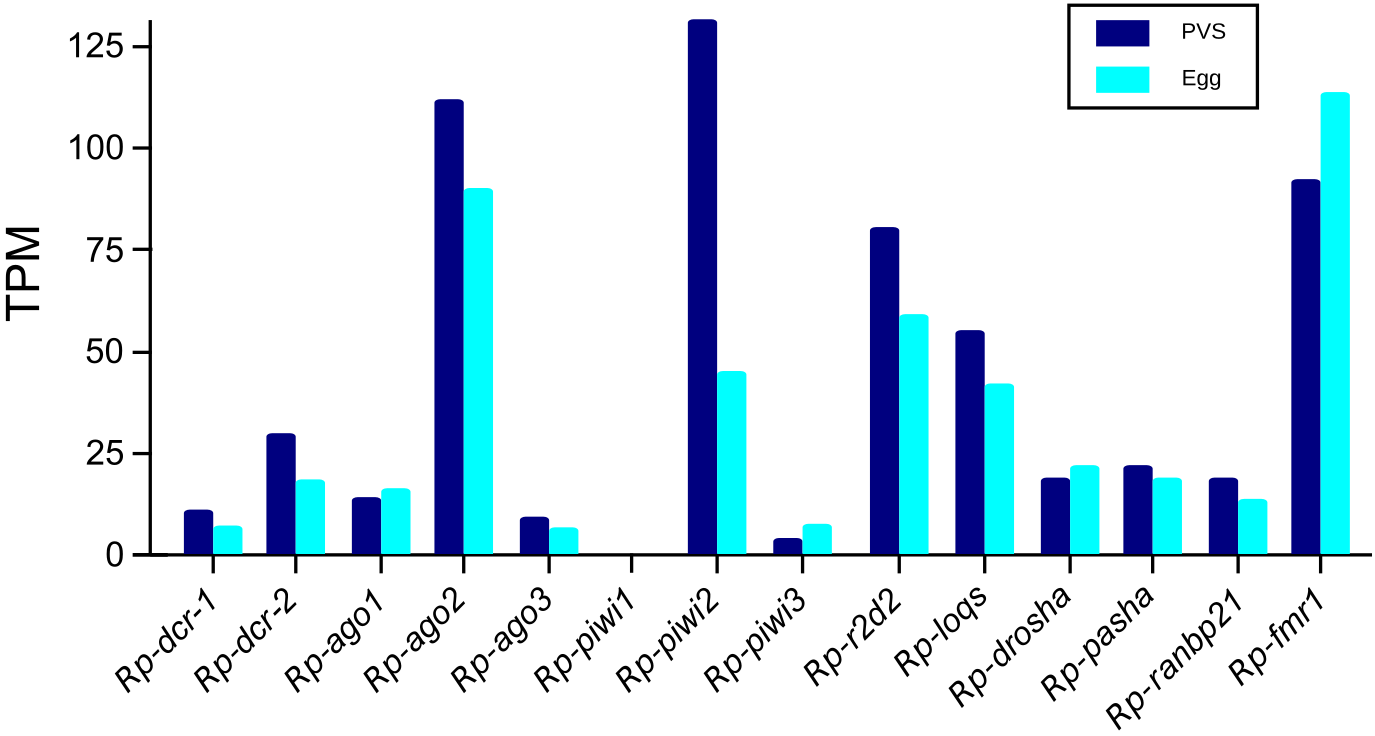

Supplement: S2 Fig — Three branches of RNAi mechanisms have been described in D. melanogaster. While the canonical RNAi is centered on Dcr2 and Ago2 proteins, Dcr1 and Ago1 are required for the miRNA pathway. Differently, the PIWI proteins Aubergine, Piwi and Ago3 act in concert with a set of other different proteins and enzymes and are key components of the piRNA pathway. Additional factors like R2D2, Loquacious, Drosha, Pasha and Exportin 5 act in different steps of the biogenesis and function of these three classes of small non-coding RNAs. R. prolixus putative orthologs of all these key proteins were identified by Blast analyses using VectorBase, FlyBase and NCBI databases. Expression levels for each gene were computed using the PVS and Egg RNA-Seq datasets. The y-axis displays Transcripts per Million (TPM). (TIFF) [file ppat.1009780.s002.tiff]

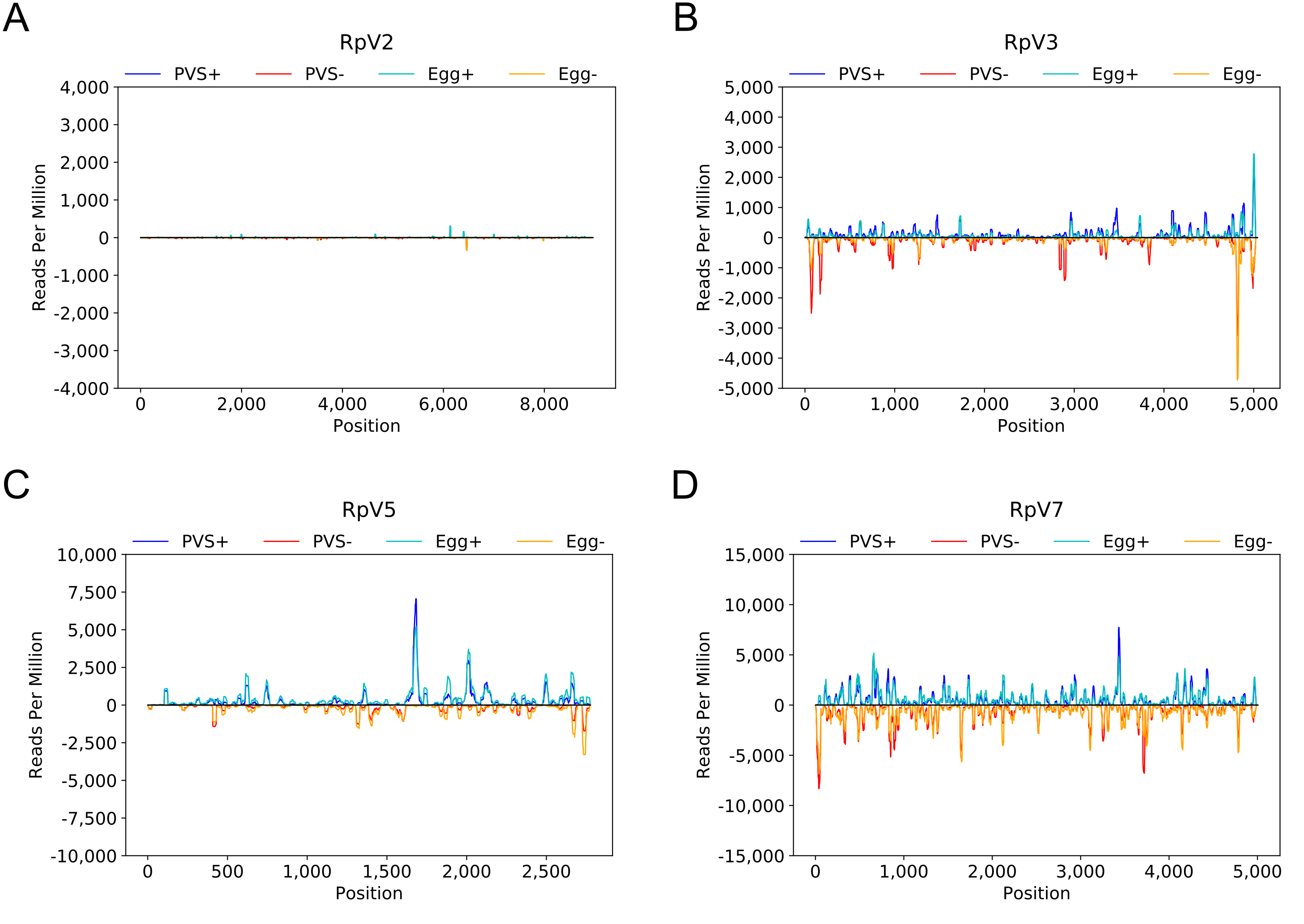

Supplement: S3 Fig — (A) RpV2, (B) RpV3, (C) RpV5, (D) RpV7. (TIFF) [file ppat.1009780.s003.tiff]

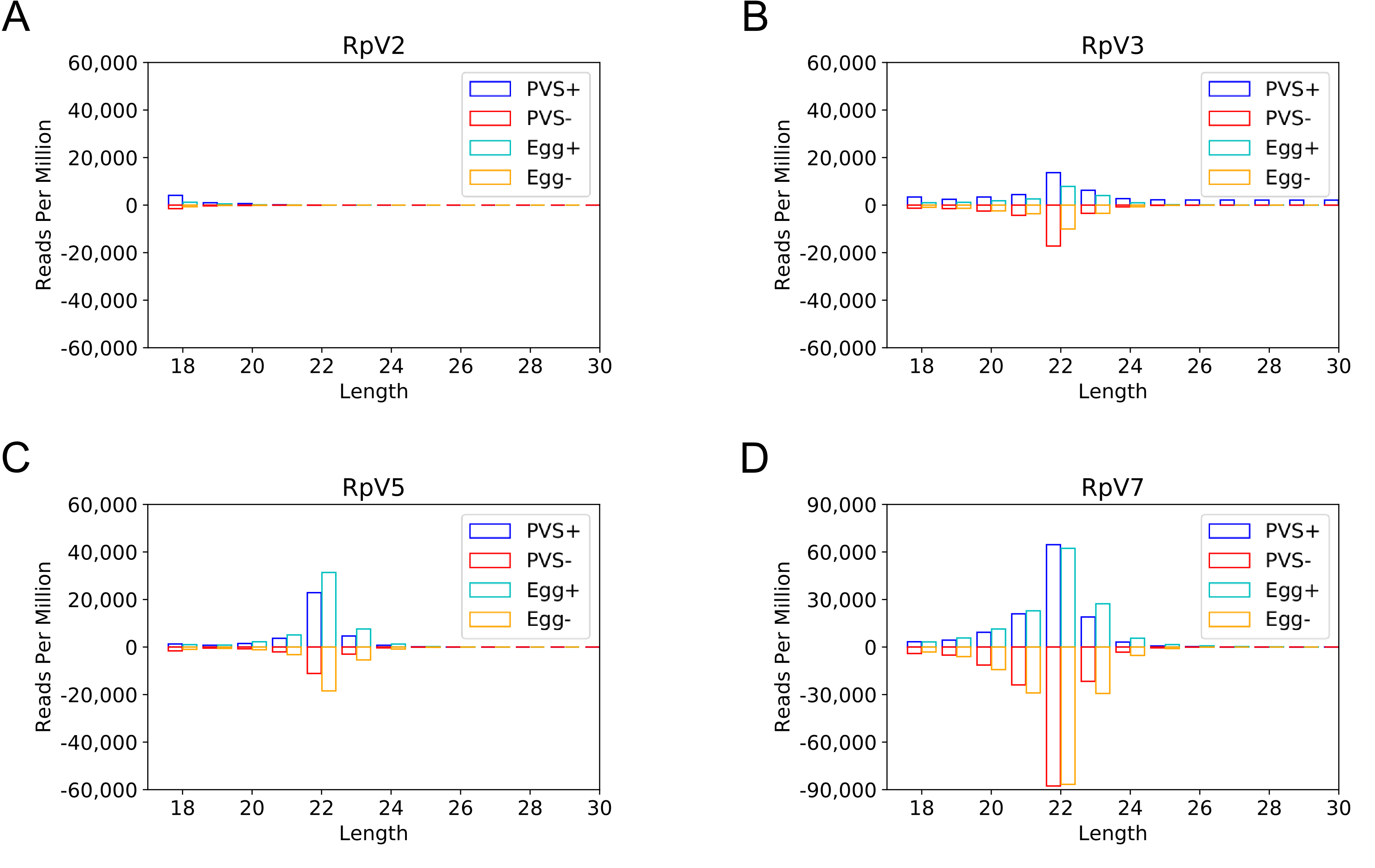

Supplement: S4 Fig — (A) RpV2, (B) RpV3, (C) RpV5 and (D) RpV7. (TIFF) [file ppat.1009780.s004.tiff]

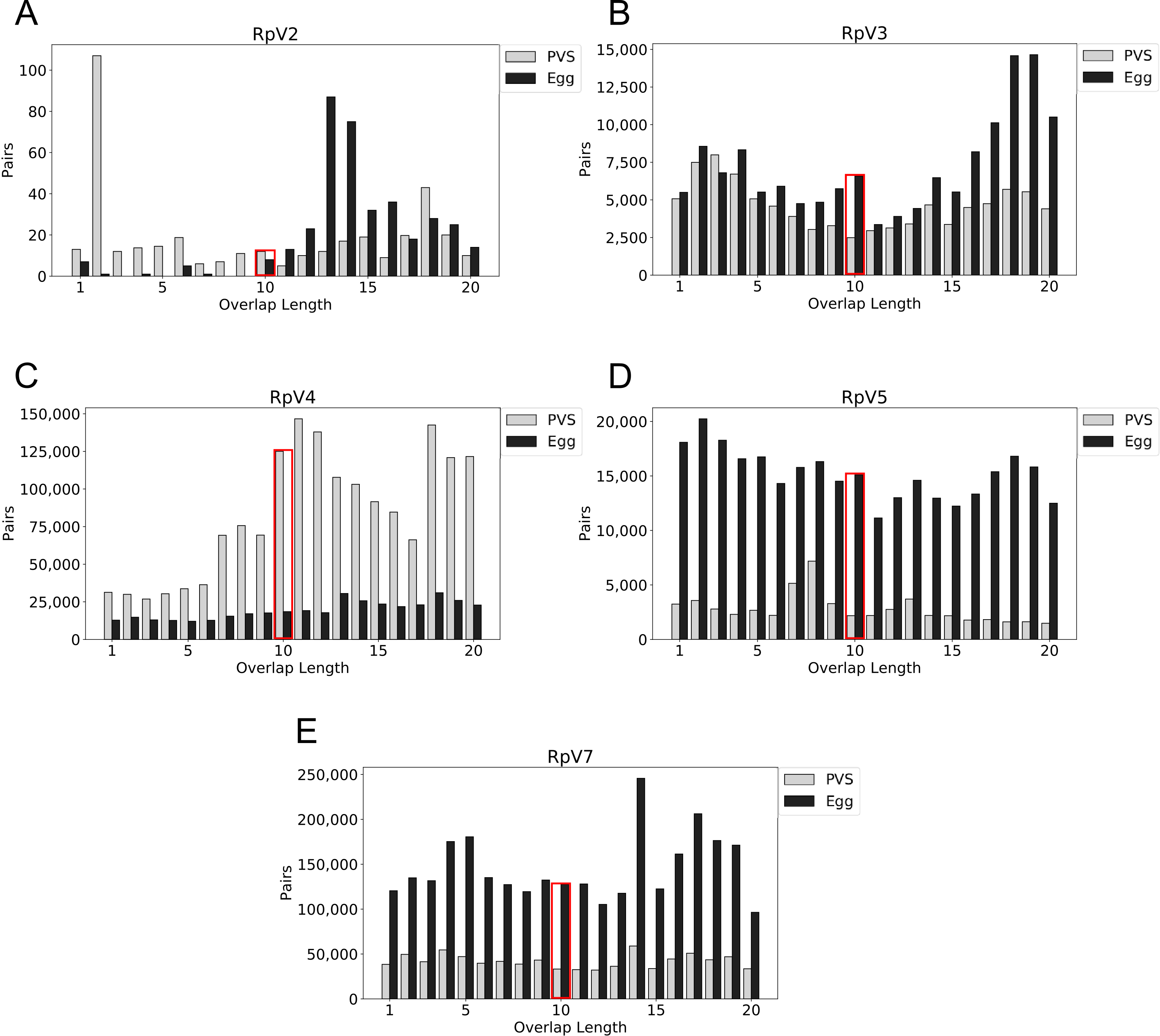

Supplement: S5 Fig — (A) RpV2, (B) RpV3, (C) RpV4, (D) RpV5 and (E) RpV7. Red boxes highlight the 10 nucleotide overlap typically found between piRNA sequences. (TIFF) [file ppat.1009780.s005.tiff]

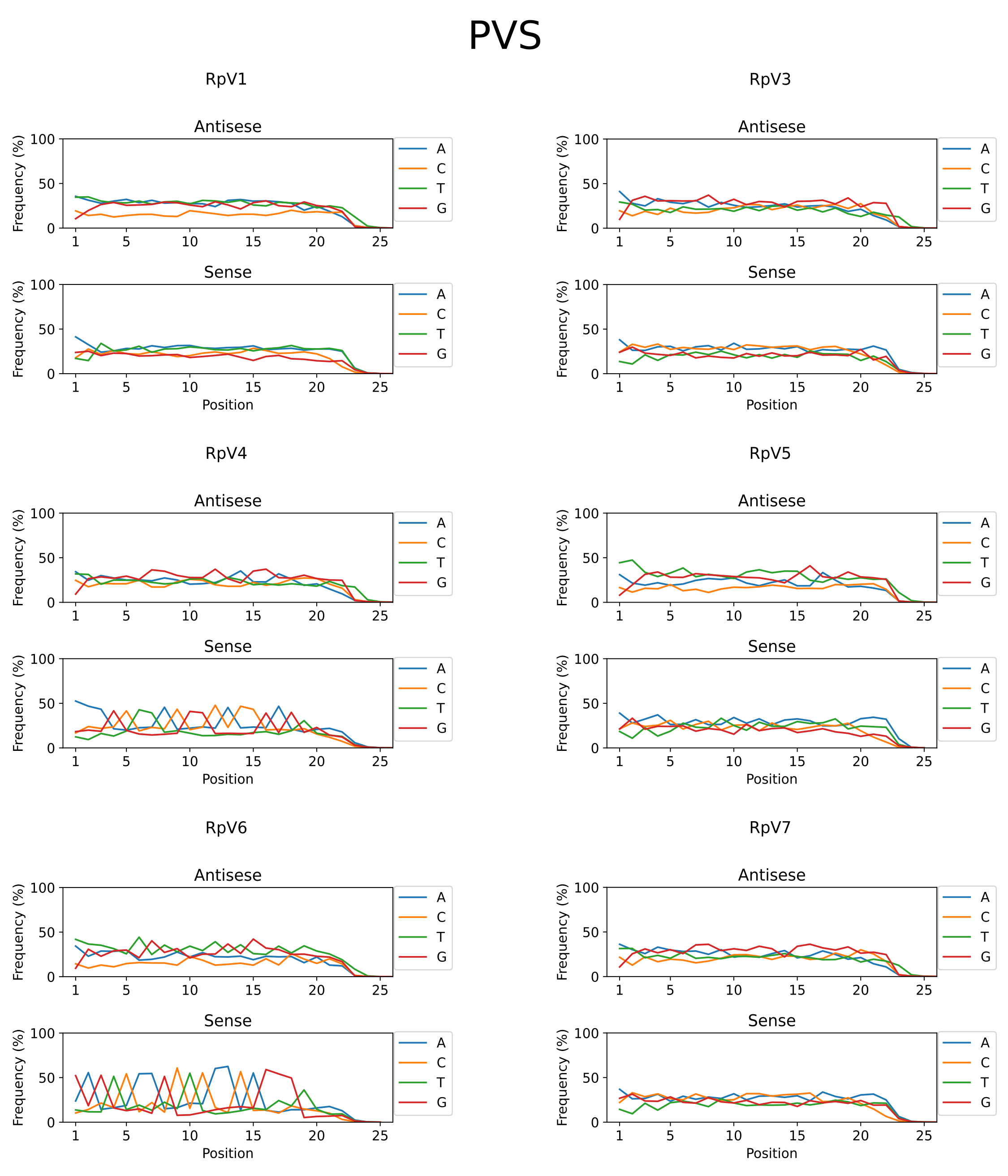

Supplement: S6 Fig — (TIFF) [file ppat.1009780.s006.tiff]

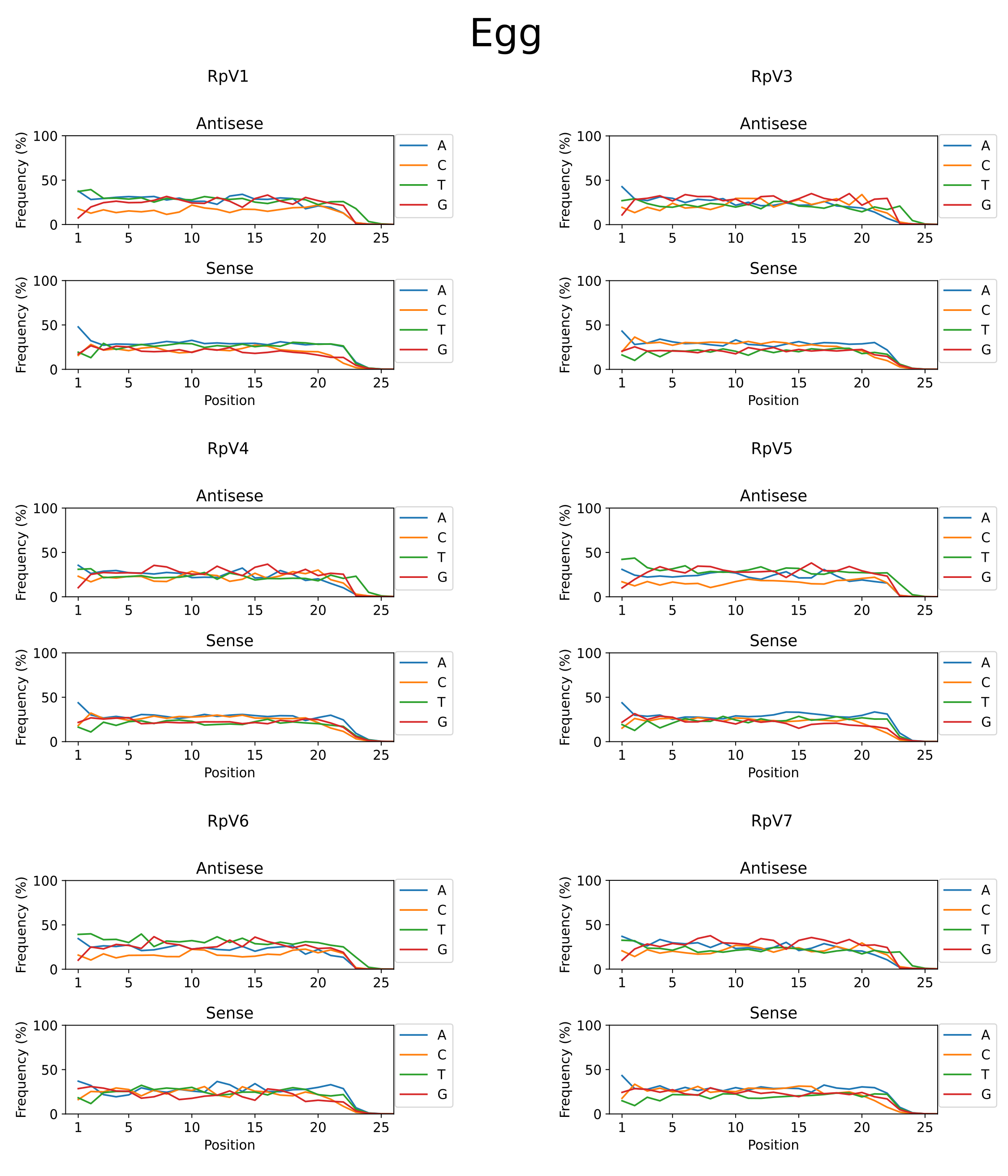

Supplement: S7 Fig — (TIFF) [file ppat.1009780.s007.tiff]

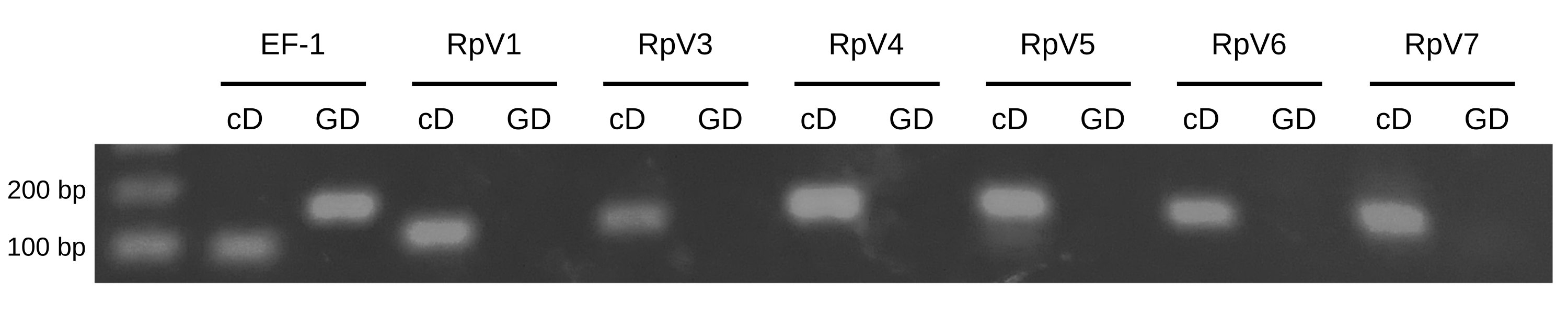

Supplement: S8 Fig — Using primers specific for each virus, we compared RT-PCR assays on ovarian cDNA (cD) for each virus with a PCR on genomic DNA (GD) extracted from Rhodnius prolixus. An amplification product of the expected length is observed for all the viruses as well as the EF-1 control gene in the RT-PCR lanes, while only EF-1 is detectable by PCR on genomic DNA. Notice that the size of the EF-1 amplification products differs between the RT-PCR and the genomic PCR due to the presence of small intron in the amplified region. The genomic DNA was extracted from 1st instar nymphs as described in Gloor et. al., 1993 [87] and oligonucleotide sequences for EF-1 were obtained from Majerowicz et. al., 2011 [88]. The oligonucleotides specific for each RpVs are those used for the qRT-PCR (Table 1). (TIFF) [file ppat.1009780.s008.tiff]

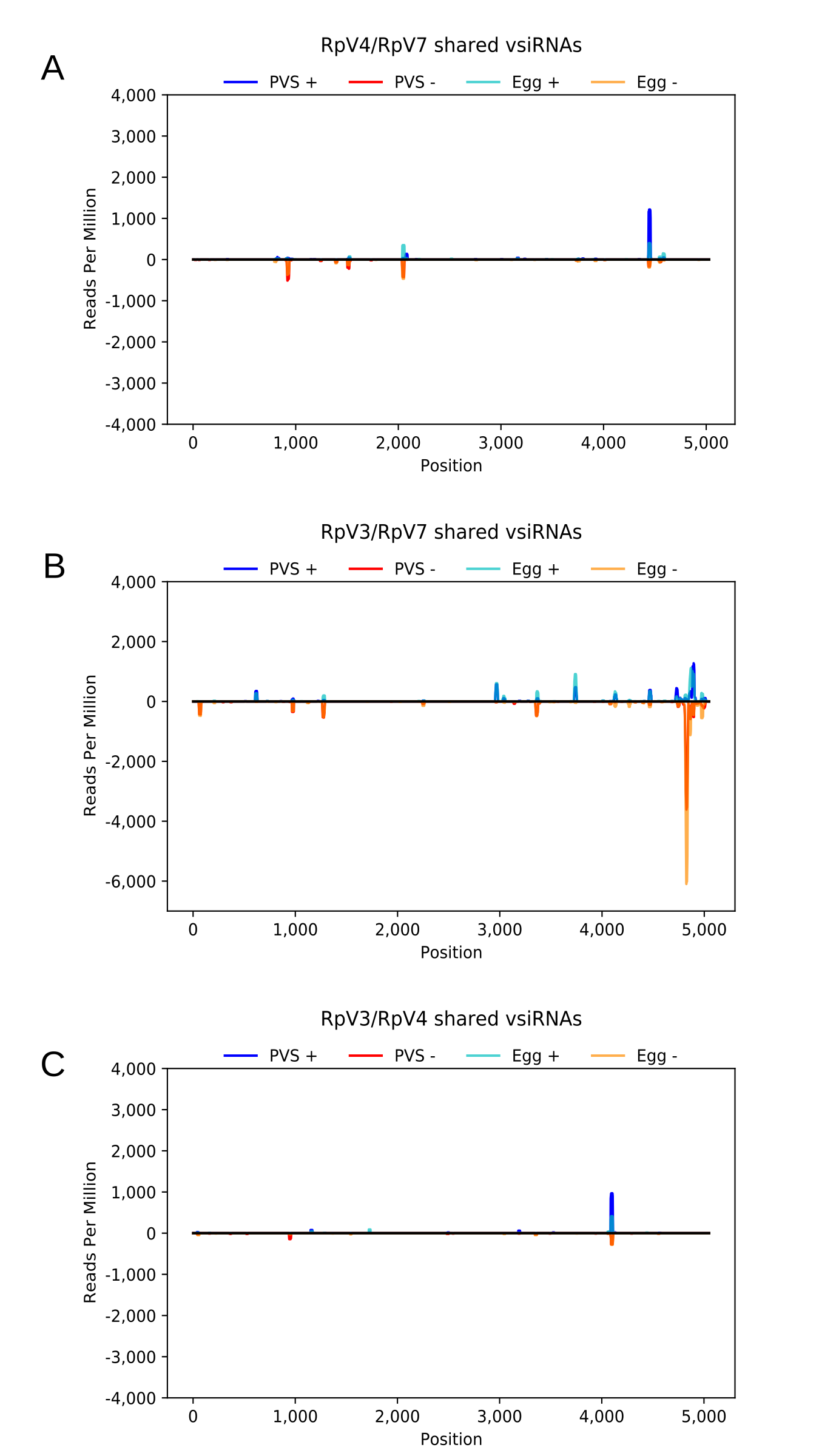

Supplement: S9 Fig — (A) RpV4 and RpV7, (B) RpV3 and RpV7, (C) RpV3 and RpV4. (TIFF) [file ppat.1009780.s009.tiff]

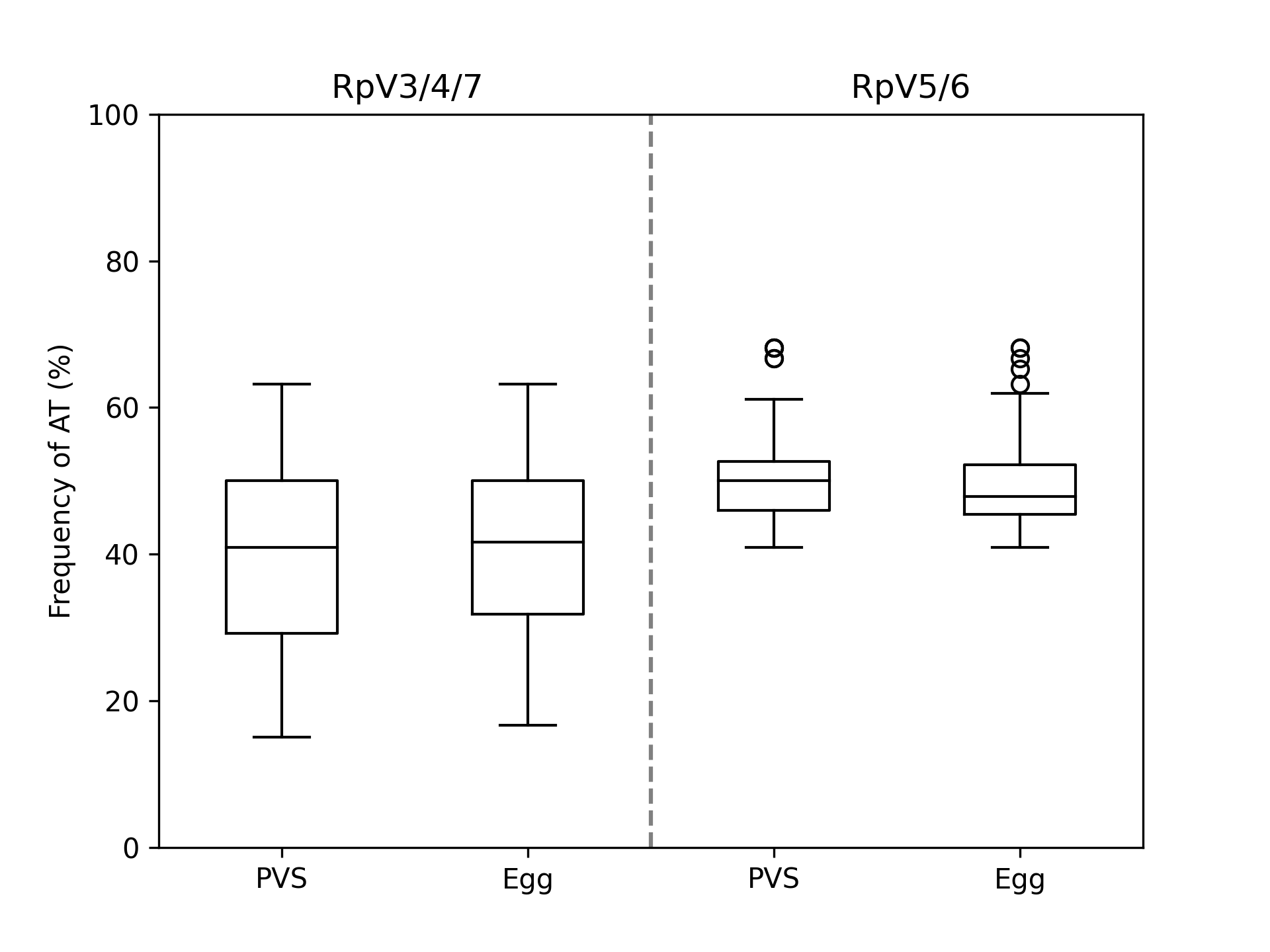

Supplement: S10 Fig — The complexity was determined by the frequency of As and Ts (y-axis) in each shared vsiRNA mapped in the 3’ UTR of the hybrid genomes in Fig 4C and 4D. None of the sequences were considered low-complexity since they did not exceed the threshold of 80% of As and Ts. (TIFF) [file ppat.1009780.s010.tiff]
